# Upper Limb Posture Estimation in Robotic and Virtual Reality-Based Rehabilitation

**DOI:** 10.1155/2014/821908

**Published:** 2014-07-08

**Authors:** Camilo Cortés, Aitor Ardanza, F. Molina-Rueda, A. Cuesta-Gómez, Luis Unzueta, Gorka Epelde, Oscar E. Ruiz, Alessandro De Mauro, Julian Florez

**Affiliations:** ^1^eHealth and Biomedical Applications, Vicomtech-IK4, Mikeletegi Pasealekua 57, 20009 San Sebastián, Spain; ^2^Laboratorio de CAD CAM CAE, Universidad EAFIT, Carrera 49 No. 7 Sur-50, 050022 Medellín, Colombia; ^3^Biomechanics, Ergonomy and Motor Control Laboratory (LAMBECOM), Physical Therapy, Occupational Therapy, Rehabilitation and Physical Medicine Department, Rey Juan Carlos University, 28922 Madrid, Spain

## Abstract

New motor rehabilitation therapies include virtual reality (VR) and robotic technologies. In limb rehabilitation, limb posture is required to (1) provide a limb realistic representation in VR games and (2) assess the patient improvement. When exoskeleton devices are used in the therapy, the measurements of their joint angles cannot be directly used to represent the posture of the patient limb, since the human and exoskeleton kinematic models differ. In response to this shortcoming, we propose a method to estimate the posture of the human limb attached to the exoskeleton. We use the exoskeleton joint angles measurements and the constraints of the exoskeleton on the limb to estimate the human limb joints angles. This paper presents (a) the mathematical formulation and solution to the problem, (b) the implementation of the proposed solution on a commercial exoskeleton system for the upper limb rehabilitation, (c) its integration into a rehabilitation VR game platform, and (d) the quantitative assessment of the method during elbow and wrist analytic training. Results show that this method properly estimates the limb posture to (i) animate avatars that represent the patient in VR games and (ii) obtain kinematic data for the patient assessment during elbow and wrist analytic rehabilitation.

## 1. Introduction

Robotic and VR technologies are important components of the modern neurorehabilitation systems for pathologies such as stroke or spinal cord injury [1–3]. In this field, our general research has two main goals:

- to improve the assessment of the rehabilitation progress through precise estimation of the patient kinematics. This is the focus of this paper;
- to optimize the rehabilitation processes by using the kinematic (and other) patient models. This optimization includes hybrid technologies (e.g., robotics, virtual reality, functional electrical stimulation [4], etc.). Even though this domain is very important for rehabilitation, we see it as a natural consequence of (a) and we concentrate on (a) at this time.

In the mentioned scenario, the proper estimation of the patient limb posture is a fundamental prerequisite for the following:

1. design and control of the advanced robotic exoskeletons which provide assistance to the patient during motor rehabilitation [5, 6],
2. animation of realistic avatars representing the patient in virtual reality (VR) scenarios (e.g., games, bionics), and
3. acquisition of kinematic data of the patient during the training exercises to assess improvement along the therapy.

This paper presents a method for estimation of limb posture from the exoskeleton posture. Notice that such an estimation is not trivial, since the limb is not rigid, is not standard, and has kinematic topology different from the exoskeleton topology.

Our method delivers limb postures estimates to strengthen and to enable downstream applications in robotic rehabilitation (among others, using VR [4]).

### 1.1. Robotic-Based Motor Rehabilitation Therapy

The inclusion of robotic devices in motor rehabilitation therapies has been increasing over the last decade. The robot-assisted therapies complement conventional rehabilitation by providing intensive, repetitive, task-specific, and interactive treatment. All these factors contribute to a more effective rehabilitation [7–9].

Robotic-assisted therapy has been shown to improve active movement, strengthening, and coordination in stroke patients [10]. The majority of clinical studies have reported that robot-assisted therapy can ease impairments and lower disabilities of the affected patient [11]. Moreover, evidence suggests that task-oriented exercises using robotic devices produce significant improvements in recovering lost abilities [12].

Combining these exercises with VR games makes the therapy more attractive to the patient, increasing motivation and treatment effects [4, 13]. It is important that these games are designed to be consistent with the principles of physical therapy and adjustable to the level of impairment [14].

A central element in designing a therapy is the feedback that patients receive. To achieve relatively permanent changes in the capability of producing skilled action, it is crucial to provide the patient with proper feedback in order to produce a positive impact on the neural mechanism promoting motor learning [15].

Feedback includes all the sensory information as the result of a movement and it is divided into two classes: (1) intrinsic or inherent feedback, which is information captured by human sensory systems as a result of the normal production of the movement, and (2) extrinsic or augmented feedback, which is information that supplements intrinsic feedback [15, 16]. Robotic-assisted therapy with VR games including animated realistic avatars may improve the quality and specificity of extrinsic feedback that the patient receives.

From the perspective of the therapist, robotic devices can be used to obtain quantitative metrics for the assessment of the improvement of the patient. The kinematic information of the affected limb during the exercises is required to compute several evaluation metrics, such as joint amplitudes, speeds, movement smoothness, and directional control.

### 1.2. Case Study Armeo Exoskeleton

**Figure 1:**
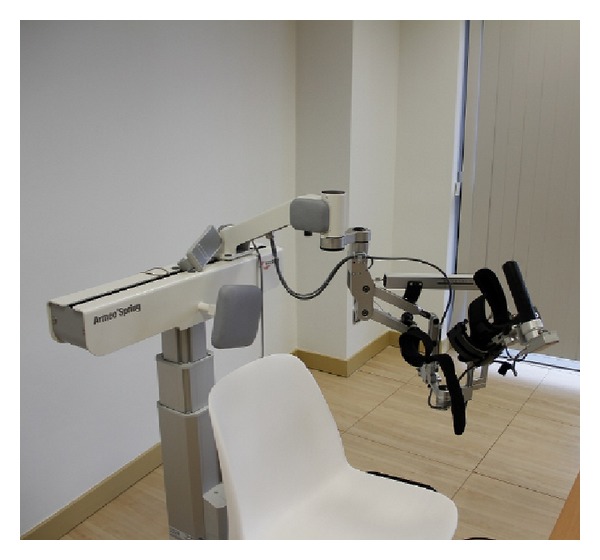
Armeo Spring orthosis.

Our proposed therapy uses the Armeo Spring exoskeleton for the upper limb intervention (Figure 1). We find the following limitations of this system.

1. Currently, the gaming platform provides an elementary assessment of the patient performance with metrics such as Hand Path Ratio [17] and joint range of motion, which are only available in certain games of the Armeo proprietary platform. We propose a continuous quantification of the patient performance along the treatment therapies, involving metrics that are highly correlated with the functional recovery of the patient.
2. Currently, the games only provide the patient with feedback of his hand position. We propose to provide a 3-dimensional representation of the arm, which would help the patient to immerse in the VR environment.

The kinematic data provided by the exoskeleton samples the angular position of its joints. Such information cannot be used directly to represent the human arm, since the patient limb and the exoskeleton kinematic models differ significantly.

This paper presents a method to estimate the posture of the limb by using the kinematic data provided by the exoskeleton. We propose to solve the limb's inverse kinematics (IK) problem extended with the kinematic constraints of the exoskeleton fixations on the limb. This extended problem is solved in real time with standard robotic libraries. In this manner, we aim to overcome the limitations of the Armeo system regarding to the feedback and assessment of the patient.

This paper is organized as follows: Section 2 presents a brief literature review.  Section 3 addresses the formal statement of the problem and the proposed method to solve it.  Section 4 discusses the implementation of our approach and its use in VR games.  Section 5 presents the evaluation methodology of our approach in the realm of motor rehabilitation.  Section 6 informs and discusses the results of the experiments conducted using our solution strategy.  Section 7 concludes the paper and identifies future developments.

## 2. Literature Review

Several estimation methods and human models have been proposed in the literature to solve the problem of limb posture estimation. Next, we present a brief review of developments in these areas.

### 2.1. Limb Posture Estimation

#### 2.1.1. Free Movement Scenario

Most of the existing work on limb posture estimation focuses on free movement scenarios. We define a free movement scenario as a situation in which the patient limb does not wear an exoskeleton or interact with any other robotic interface. Under the mentioned conditions, the literature that addresses upper limb posture estimation considers tasks in which the human subject has to reach a desired object. Therefore, these approaches are designed to estimate the posture of the upper limb based on a given target position and orientation of the hand.

Statistical [18, 19], IK [20–22], and direct optimization [23–28] methods are the most used approaches to estimate the limb posture [29].

Statistical or data-based approaches model the human kinematics with regressive models from empirical data [30]. Factors such as the size of the database of captured motions [31] and the characteristics of the population involved in the experiments impact the accuracy and usefulness of these models.

Kinematic approaches model the human limbs with links, joints of different degrees of freedom, and end-effectors [27]. The IK problem is then solved with either closed-form or numerical methods. The quality of the kinematic model and the convergence speed and robustness of the approach used to solve the IK problem directly affect the accuracy of the estimations.

Optimization approaches require a nontrivial function to minimize, which actually leads to the desired configuration (typically, a minimal energy one [31]). When optimization is used to solve an IK problem, additional constraints can be easily included in the formulation [26–28].

Approaches combining optimization-based and statistical models have been also proposed to overcome the individual limitations of optimization and statistical methods [31, 32]. Naturally, the composed method requires a high-quality dataset of motions and the formulation of proper objective and constraints functions.

#### 2.1.2. Robotic-Assisted Scenario

There is a shortage in the literature addressing posture estimation of the human limb while interacting with an exoskeleton. Although exoskeletons are designed with the ultimate goal of minimizing their kinematic differences with human limbs and interact seamlessly with them, the following factors influence the human motion patterns and therefore the posture of the limb:

1. the mechanic design of the exoskeleton (inertia, back drivability, friction, joint motion limits, etc.).
2. the type of assistance that the exoskeleton provides (passive, active, and assist-when-needed).
3. the performance of the exoskeleton motion controller. Here, using a naive one-to-one mapping between the joint angles of the human limb and exoskeleton leads to poor positioning results [33].

References [6, 21] propose the computation of the arm's IK by using a disambiguation criteria for its redundancy which chooses a swivel angle such that the palm points to the head region. This methodology is suitable for real-time implementation and it is used in the control strategy of the active 7-DOF exoskeleton developed by the authors' research team [34]. The authors report that the mean error in the estimation of the swivel angle is less than 5 degrees. The magnitude of the errors in the estimation of the wrist, elbow, and GH-joint angles is not reported.

References [6, 21] do not consider the motions of the clavicle and scapula (which affect the position of the GH-joint center) in the estimation of the posture of the arm, as they assume the position of the GH-joint center to be known. Therefore, this approach should not be used in cases in which the position of the GH-joint center cannot be determined from data provided by the exoskeleton (e.g., Armeo Spring) or by any additional motion capture system.

Other common methods to estimate the posture of human limbs cannot be used or are impractical in robotic-assisted scenarios. For example, inertial and magnetic measurement systems (IMMSs) presented in [35, 36] are unusable because the magnetic disturbances produced by the metallic components of the exoskeleton corrupt the magnetic sensor measurements.

If optical tracking systems are used, arrays of markers need to be attached to the patient in order to measure the limb joint angles. Occlusions of such markers are frequently produced by the mechanic structure of the exoskeleton when performing the rehabilitation exercises. To overcome the occlusions of the markers, a redundant setup is necessary [29]. This limitation makes the use of optical tracking systems cumbersome for frequent use in the rehabilitation therapy.

### 2.2. Human Model

A central element in human posture estimation is the human kinematic model itself. Simple models based on hierarchies of links and lower kinematic pairs can be found in [27, 37–40]. These approaches results are convenient for real-time tasks and for implementation. However, more elaborated models should be used to describe complex kinematic relationships [41], such as the shoulder rhythm [42]. On the other hand, musculoskeletal models reported in [43–45] offer better accuracy for dynamics computations, since they include forces from muscles and ligaments.

The selection of the human kinematics model rests not only on the kinematic statement of the problem, but also on the compromise between accuracy and speed required in a particular application.

### 2.3. Conclusions of Literature Review

Although the methods designed to estimate the posture of the upper limb (in absence of a robotic interface) reviewed in Section 2.1.1 could be used in robotic-assisted rehabilitation, we have not found any actual implementation of them in this context. Usage of these methods without any change in their design parameters in robotic-assisted applications may lead to erroneous posture estimations, given the influence of the exoskeleton on human motion patterns. Therefore, the validity of these methods in the robotic-assisted scenario remains to be proven. An additional limitation of these methods is that only few of them have been validated quantitatively by determining the errors in their estimations.

On the other hand, the few posture estimation approaches that address limb interaction with an exoskeleton (Section 2.1.2) have been designed to specifically solve the arm posture estimation problem, limiting their usability in posture estimation of other human limbs.

In response to the mentioned issues, in this paper we present the following:

1. a method that can be applied, in a general manner, to solve the limb posture estimation problem using kinematic data provided by the exoskeleton attached to the limb,
2. the implementation of our proposed method for the upper limb posture estimation using the Armeo Spring exoskeleton, and
3. the quantitative validation of our proposed method by determining the estimation errors during the training of meaningful upper limb rehabilitation exercises.

## 3. Materials and Methods

### 3.1. Problem Description

In this section, we state the problem of estimating the joint angles of the patient limb during robotic-assisted rehabilitation therapy from the kinematic information provided by the robot. The elements that are considered inputs to the problem are the following: (1) the geometry and topology (e.g., the Denavit-Hartenberg parameters [46]) of the exoskeleton and the human limb, (2) a known configuration of the angles of the joints of the exoskeleton, (3) the kinematic constraints imposed by the fixations of the exoskeleton over the patient limb (which result from wearing the exoskeleton), and (4) the constraints that govern the posture of the patient limb while interacting with the exoskeleton, which are related to mechanical and control factors of the exoskeleton that influence the patient movement. The goal of the proposed algorithm is to find the approximate joint angles of the patient limb, such that the mentioned constraints are met.

This problem can be formally stated as follows.

*Given*

1. the kinematic model of the exoskeleton *R*(*L*_*R*_, *J*_*R*_), where *L*_*R*_ and *J*_*R*_ are sets of links and joints, respectively,
  - *L*_*R*_ = {*l*_*R*_0__,…, *l*_*R*_*f*+1__};
  - *J*_*R*_ = {*j*_*R*_0__,…, *j*_*R*_*f*__}:
    - *N*(*j*_*R*_*i*__) denotes the degrees of freedom (DOF) of *j*_*R*_*i*__;
    - *v*_*R*_*i*__ = {*θ*_1_,…, *θ*_*N*(*j*_*R*_*i*__)_} is a vector that contains the angles of each DOF of *j*_*R*_*i*__(*i* ∈ [0, *f*]);
  - *R* is an open kinematic chain. Therefore, *l*_*R*_*i*__ and *l*_*R*_*i*+1__ are connected by joint *j*_*R*_*i*__, where *i* ∈ [0, *f*];
  - the vector *q*_*R*_ ∈ *R* ^*n*^, *n* = ∑_*i*=0_^*f*^ *N*(*j*_*R*_*i*__), contains the set of independent coordinates that defines a configuration of *R* uniquely:
    - *q*_*R*_ = {*v*_*R*_0__,…, *v*_*R*_*i*__,…, *v*_*R*_*f*__};
    - *q*_*R*_*t*__ represents the state of *q*_*R*_ in instant *t* and its value is known;
2. a human patient with a kinematic model of his limb *H*(*L*_*H*_, *J*_*H*_), where *L*_*H*_ and *J*_*H*_ are sets of links and joints, respectively,
  - *L*_*H*_ = {*l*_*H*_0__,…, *l*_*H*_*g*+1__};
  - *J*_*H*_ = {*j*_*H*_0__,…, *j*_*H*_*g*__}:
    - *N*(*j*_*H*_*i*__) denotes the DOF of *j*_*H*_*i*__;
    - *v*_*H*_*i*__ = {*θ*_1_,…, *θ*_*N*(*j*_*H*_*i*__)_} is a vector that contains the angles of each DOF of *j*_*H*_*i*__  (*i* ∈ [0, *g*]);
  - *H* is an open kinematic chain. Therefore, (*l*_*H*_*i*__) and (*l*_*H*_*i*+1__) are connected by joint (*j*_*H*_*i*__), where *i* ∈ [0, *g*];
  - the vector *q*_*H*_ ∈ *R* ^*k*^, *k* = ∑_*i*=0_^*g*^ *N*(*j*_*H*_*i*__), contains the set of independent coordinates that defines a configuration of *H* uniquely:
    - *q*_*H*_ = {*v*_*H*_0__,…, *v*_*H*_*i*__,…, *v*_*H*_*g*__};
    - the *i*th element of *q*_*H*_, *θ*_*i*_, is subject to *h*_*i*_(*θ*_*i*_) = *θ*_min⁡_*i*__ ≤ *θ*_*i*_ ≤ *θ*_max⁡_*i*__  (*i* ∈ [0, *k* − 1]);
    - *q*_*H*_*t*__ represents the state of *q*_*H*_ in instant *t* and its real value is unknown;
3. a set of passive mechanisms *M* = {*m*_0_,…, *m*_*p*_} that connect *R* and *H*:
  - *m*_*i*_  (*i* ∈ [0, *p*]) connects *l*_*R*_*a*__  (*a* ∈ [0, *f* + 1]) and *l*_*H*_*b*__  (*b* ∈ [0, *g* + 1]);
  - *m*_*i*_ imposes a movement constraint of *N*(*m*_*i*_)-DOF to *l*_*H*_*b*__ with respect to *l*_*R*_*a*__;
  - the set *C*(*M*) = {*c*_0_,…, *c*_*p*_} contains vector-valued functions *c*_*i*_(*q*_*H*_*t*__, *q*_*R*_*t*__) ∈ *R* ^*N*(*m*_*i*_)^  (*i* ∈ [0, *p*]) that model the kinematic constraint imposed by *m*_*i*_;
  - each *c*_*i*_(*q*_*H*_*t*__, *q*_*R*_*t*__) is an equality constraint of the form *c*_*i*_(*q*_*H*_*t*__, *q*_*R*_*t*__) = 0;
4. a set of vector-valued constraint functions *D* = {*d*_0_,…, *d*_*s*_} that intend to represent the performance measures that govern the posture of the limb in a specific situation:
  (a) each *d*_*i*_(*q*_*H*_*t*__) (*i* ∈ [0, *s*]) is an equality constraint of the form *d*_*i*_(*q*_*H*_*t*__) = 0;
  (b) the dimension of the *d*_*i*_ vector is denoted by dim⁡(*d*_*i*_).

*Goal is as follows*

(1) to find the vector ${\tilde{q}}_{H_{t}}\in R^{k}$, which approximates *q*_*H*_*t*__ such that
  - *c*_*i*_(*q*_*H*_*t*__, *q*_*R*_*t*__) = 0  ∀ *i* ∈ [0, *p*];
  - *h*_*j*_(*θ*_*j*_) = *θ*_min_*j*__ ≤ *θ*_*j*_ ≤ *θ*_max_*j*__  ∀ *j* ∈ [0, *k* − 1];
  - *d*_*u*_(*q*_*H*_*t*__) = 0  ∀ *u* ∈ [0, *s*].

To solve this problem, a method based on IK of the limb has been developed. The following sections describe the methodology implemented.

### 3.2. Kinematic Modeling of the Exoskeleton

The Armeo Spring (Figure 1) is a passive exoskeleton (orthosis) that supports the weight of the arm of the patient. The level of support provided by the system springs can be adjusted, regulating the effort of the patient arm to overcome gravity. The exoskeleton has a total of seven angle sensors to measure the position of its rotational joints and one pressure sensor to measure the gripping force at the hand [47].

**Figure 2:**
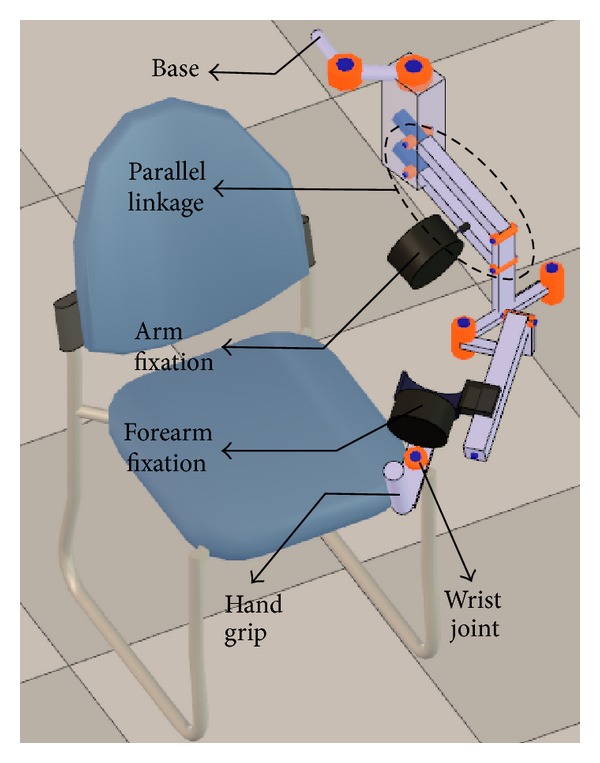
Exoskeleton kinematic model.

We built a kinematic model of the Armeo Spring (Figure 2), which contains both prismatic and revolute joints. The prismatic joints of the exoskeleton allow adjusting it to the different sizes of the patients, and they remain fixed during the training.

Our implementation models the links and joints of the Armeo exoskeleton and creates a hierarchical structure of them.

Although the Armeo exoskeleton presents a parallelogram mechanism in its kinematic chain, the exoskeleton can be modeled with a serial chain extended with a dependency equation among the joints used to represent the parallel mechanism.

### 3.3. Kinematic Modeling of the Human Upper Body

**Figure 3:**
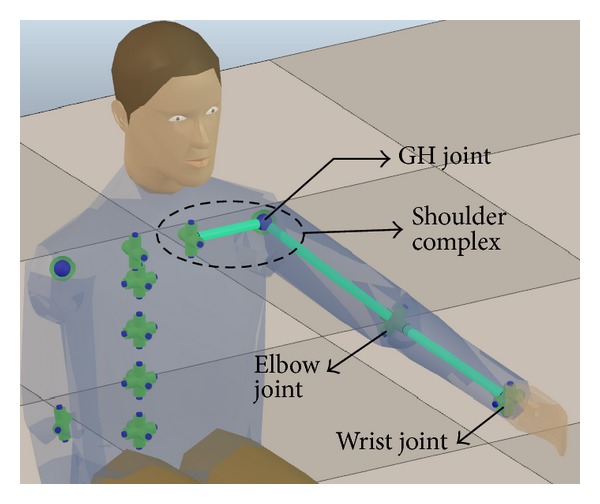
Human upper body kinematic model.

Figure 3 shows the kinematic model of the human upper body that we created for this application. The joints of the model are represented with green color. The upper limb is highlighted using links in light green color.

Our upper body model (33-DOF) includes joints of the spine, shoulder complex, elbow, and wrist. It is based on the ones presented in [27–29, 38, 39, 48], which have been widely used in the area of human posture estimation. The main advantages of those models are their easy implementation and their suitability for solving the posture estimation problem in real time, which is one of the main requirements of our application. A weakness of those kinematic models is that the glenohumeral (GH) joint is modeled with a kinematic chain of three concurrent revolute joints, orthogonal to each other. In this way, the rotation of the GH joint is parameterized with Euler angles and suffers from gimbal lock [49]. In order to avoid this limitation, the GH joint is represented in our model with a spherical joint, such that other rotation parameterizations (e.g., quaternion or exponential map) can be used.

Although there are more complex and accurate kinematic models of the upper body, the results obtained in [39], in a scenario where the subject does not interact with an exoskeleton in an application that is not related to motor rehabilitation, show that posture estimations for the upper limb can be obtained with a reasonable accuracy by using their original model.

The neutral or rest posture of the arm is defined with the arm fully extended along the body as in [50]. The range of motion of the joints of the arm obtained in [34] (derived from a motion study during the execution of activities of daily living) is used as reference to establish the joint limits of our model, which correspond to constraint 2(d)(ii) in the list presented in Section 3.1.

### 3.4. Modeling the Kinematic Constraints of Interaction of the Upper Limb and the Exoskeleton

The Armeo provides fixations for the human limb. These fixations introduce constraints on the position and orientation of the coordinate systems attached to the arm, forearm, and hand.

There are several factors that affect the satisfaction of the constraints during the execution of the exercises. This set includes (1) deformation of the coupling mechanisms and (2) uncertainty or errors in the modeling of the human upper limb. Therefore, these constraints are exactly met only under ideal conditions and in practice they do not capture all the details of the real interaction. However, as we prove, they suffice to obtain a reasonable accuracy in the estimation of the limb posture.

#### 3.4.1. Arm Constraint

The arm fixation imposes a position (3-DOF) constraint on the human arm. The point on the arm that follows the position of the fixation is determined by an initialization process between the *R* and *H* kinematic chains (see Section 3.6).

In our model, the fixations are modeled as rigid bodies. However, the exoskeleton fixations are made of flexible materials, such that their geometry is deformed when large forces are applied on them.

The arm fixation suffers significant deformation when the arm is moved towards a horizontal configuration (e.g., when performing a complete stretching of the arm along the sagittal or frontal plane). In those cases, the coordinate system at the exoskeleton arm fixation center undergoes a translation, resulting from the deformation of the fixation mechanism that is not reproduced by our model.

To deal with this kind of situations, the weights of constraints representing fixations that suffer less deformation than other ones are adjusted such that they receive more importance when solving the IK problem. In this way, the limb posture is estimated meeting the constraints that model with more fidelity the observed behavior. In this case, the weight of the arm constraint is lower than the ones belonging to the forearm and arm restrictions.

**Figure 4:**
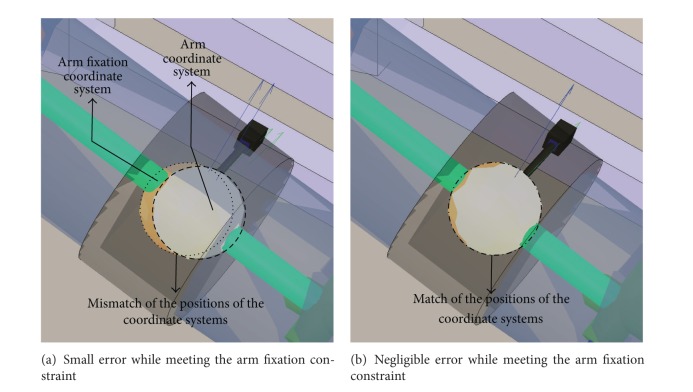
Constraint modeling the interaction of the Armeo's arm fixation.

Figure 4 shows the human arm (blue transparent cylinder) with the fixation of the exoskeleton for the arm (black transparent ring) around it. The constraint imposed by this fixation to the arm is represented by the matching of (a) human arm (white disk) versus (b) fixation (yellow disk) coordinate systems. Figures 4(a) and 4(b) correspond to unsatisfied and satisfied constraints, respectively.

#### 3.4.2. Forearm Constraint

The forearm fixation imposes a 3-DOF position constraint on the human forearm. The point on the human forearm that moves together with the fixation is determined in the initializing stage. Additionally, the fixation is able to rotate around its longitudinal axis, according to the forearm pronation/supination movement (1-DOF orientation constraint). The rotation angle is measured with an encoder. The forearm constraint forces the human wrist flexion/extension axis to be approximately aligned with the exoskeleton's wrist joint axis.

#### 3.4.3. Hand Constraint

The hand constraint forces the human hand to follow the position and orientation (6-DOF) of the Armeo hand grip. The patient exercises while grabbing the handle of the exoskeleton. The mechanic design of the Armeo avoids the slippage of the hand with respect to the axis of the handle during the execution of the exercises. As with the previous fixations, the point on the hand where the coordinate system of the hand is located is calculated in the initialization stage.

#### 3.4.4. Shoulder Constraint

The shoulder constraint does not belong to the set of movement restrictions imposed by the coupling mechanisms of the Armeo. Instead, it is related to the restrictions intended to produce a natural posture of the upper limb considering also the influence of the exoskeleton on the patient movements. This constraint helps to choose one of the multiple configurations of the human kinematic chain that comply with the other categories of constraints.

Currently, it is implemented to attract the GH joint to a position (3-DOF position constraint) below the first joint of the Armeo (*j*_*R*_0__ joint represented with symbol *A* in Figure 6), which does not suffer any translation during the training of the patient. By keeping the GH joint near *j*_*R*_0__ comfortable postures for the spine and arm can be achieved.

**Figure 5:**
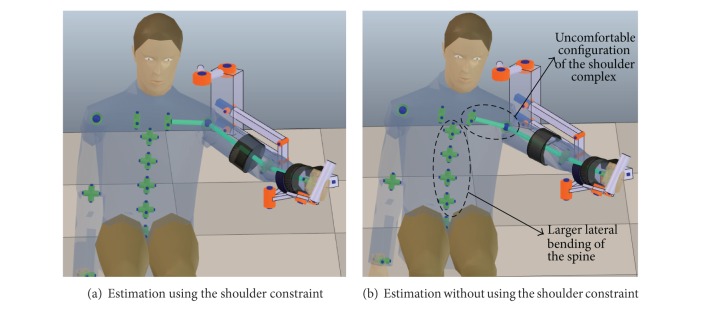
Effect of the shoulder constraint in the upper limb posture estimation.

Figure 5 shows that the shoulder constraint prevents the excessive motion of the joints of the spine, which is a compensatory movement that should be also avoided during the rehabilitation therapy. The shoulder constraint is central to proper posture estimation during shoulder abduction.

### 3.5. Inverse Kinematics

Given a desired pose (position and orientation) vector *T*_*r*_ ∈ *R* ^6^ for the end-effector of an open kinematic chain *r*, the IK problem is to find the vector of angles of the robot's joints *q*_*r*_ ∈ *R* ^*N*^ (where *N* corresponds to the DOFs of *r*), such that the difference *e* = *T*_*r*_ − *X*_*r*_ between *T*_*r*_ and the actual pose of the end-effector of *r*, *X*_*r*_ ∈ *R* ^6^, approaches zero.

There are several approaches to solve this problem, including analytic [51] and numerical methods [52, 53]. The iterative strategy used to solve the IK problem is based on the Jacobian matrix of the manipulator *Z*(*q*_*r*_), which linearly relates the velocity of the end-effector and the joints by

(1) $$\begin{array}{c} {\dot{X}}_{r}=Z\left(q_{r}\right){\dot{q}}_{r}. \end{array}$$

By replacing Δ*X*_*r*_ for *e* in (2), which is obtained by discretizing (1), the necessary Δ*q*_*r*_ to approximate *T*_*r*_ is obtained:

(2) $$\begin{array}{c} \Delta q_{r}=Z{\left(q_{r}\right)}^{-1}\Delta X_{r}. \end{array}$$

Notice that *Z*(*q*_*r*_) may not be square (consider, e.g., a kinematic chain with more than 6-DOF) or invertible. In those cases, the pseudoinverse and damped least squares (DLS) methods (among others) can be used to obtain Δ*q*_*r*_, such that ||*e*|| is minimized. The pseudoinverse method is computationally faster than the DLS but tends to be unstable when the robot approaches a singular configuration. The DLS method offers more robustness (specially when *T*_*r*_ is out of reach) at the cost of a slower convergence [52].

#### 3.5.1. Relation among End-Effectors and Targets

The aforementioned strategy to solve the IK problem can also be used in situations in which the manipulator has more than one end-effector. In this case, the error vector *e* is given by *e* = {*T*_*r*_1__ − *X*_*r*_1__,…, *T*_*r*_*i*__ − *X*_*r*_*i*__,…, *T*_*r*_Nee__ − *X*_*r*_Nee__} where Nee is the number of end-effectors of the robot. Notice that vector *e*_*i*_ = *T*_*r*_*i*__ − *X*_*r*_*i*__ is not necessarily a point ∈*R* ^6^. For example, if only the position (and not the orientation) of the *i*th end-effector is specified, *e*_*i*_ ∈ *R* ^3^.

In our application, the formulation of the IK problem with multiple end-effectors and targets can be used to represent the constraints discussed in Section 3.4. In this way, each constraint can be represented by a target and end-effector pair. The coordinate frames of the end-effectors *X*_*H*_*i*__(*q*_*H*_*t*__)  (*i* ∈ [1,…, Nee]) are attached to the human limb, so their position and orientation depend on the current configuration of the limb, *q*_*H*_*t*__. The coordinate frames of the targets of the limb *T*_*H*_*i*__(*q*_*R*_*t*__)  (*i* ∈ [1,…, Nee]) are attached to the exoskeleton such that they are transformed according to its current configuration *q*_*R*_*t*__. Then, the IK problem is solved for the limb, finding *q*_*H*_*t*__ such that *e*_*i*_ = ||*T*_*H*_*i*__(*q*_*R*_*t*__) − *X*_*H*_*i*__(*q*_*H*_*t*__)|| ≈ 0  (*i* ∈ [1,…, Nee]). Notice that if *e*_*i*_ represents a kinematic constraint, *e*_*i*_ ∈ *R* ^*N*(*m*_*i*_)^ where *i* ∈ [0, *p*]. Otherwise, *e*_*i*_ represents a restriction related to the natural posture of the limb, and therefore *e*_*i*_ ∈ *R* ^dim⁡(*d*_*i*_)^, where *i* ∈ [0, *s*], and Nee = *p* + *s* + 2.

Notice that, due to modeling inaccuracies of the kinematic chains or the constraints, it is possible that for a configuration *q*_*R*_*t*__ some constraints cannot be satisfied within the desired tolerance. That situation can be interpreted as if some targets *T*_*H*_*i*__(*q*_*R*_*t*__) are not reachable. It is important that the method used to solve the IK problem handles this situation robustly, avoiding oscillations. For this reason the DLS method was used.

#### 3.5.2. Joints and Constraint Weights

References [38, 39] state that giving more importance to some of the model joints over others, by assigning weights to the joints, allows estimating more accurately the posture of the human limb.

Let us assume that *w*_*J*_*i*__ is the weight of joint *J*_*H*_*i*__ and that joints *J*_*c*_ and *J*_*d*_ can contribute to the movement of end-effector *i* to diminish *e*_*i*_. Then, if *w*_*J*_*c*__ > *w*_*J*_*d*__, the displacement that *J*_*c*_ performs is larger than the one done by *J*_*d*_. This means that *J*_*c*_ is preferred to be moved over *J*_*d*_ to reach a desired target.

In our model, the weights of the joints of the upper body were adjusted such that the joints on the spine of the model perform small displacements in comparison with the movement performed by the shoulder, elbow, and wrist joints.

On the other hand, applying weights to the error vector *e* gives more importance to reach a specific target over others. In our approach, this translates into giving some constraints more importance than others. Let us define with *w*_*c*_*i*__  (*i* ∈ [0, *p*]) the weight of the *c*_*i*_ constraint and with *w*_*d*_*u*__  (*u* ∈ [0, *s*]) the weight of *d*_*u*_ constraint.

In our model, high weights were adjusted for the kinematic constraints imposed by the exoskeleton fixations (*w*_*c*_*i*__ ≈ 1.0). Otherwise, low weights (*w*_*d*_*i*__ ≈ 0.2) were assigned to the other type of constraints.

There are different formulations of the DLS method that incorporate weights for the joints and error vector (e.g., [54]). In V-REP, the following DLS formulation is used to solve IK problems. The angles of the joints of the human model are given by $q_{H_{t}}=\sqrt{W_{q}}q_{H_{t_{w}}}$, where *q*_*H*_*t*_*w*___ = *Z*_w_*e_*w*_ and *Z*_w_* = *Z*_*w*_^*t*^(*Z*_*w*_ *Z*_*w*_^*t*^ + *αI*)^−1^. The weighted Jacobian matrix is given by $Z_{w}=Z\sqrt{W_{q}}$, where *W*_*q*_ = diag⁡{*w*_0_,…, *w*_*k*−1_}. Here, if *w*_*a*_ and *w*_*b*_ are related to *J*_*H*_*i*__ (e.g., a joint with DOFs > 1), *w*_*a*_ = *w*_*b*_ = *w*_*J*_*i*__. The weighted error vector is given by *e*_*w*_ = *W*_*e*_ *e*, where *W*_*e*_ = diag⁡{*w*_0_,…, *w*_*v*−1_} and *v* = ∑_*i*=0_^*p*^ *N*(*m*_*i*_) + ∑_*j*=0_^*s*^dim⁡(*d*_*j*_). If *w*_*a*_ and *w*_*b*_ are related to the same *c*_*i*_ constraint, *w*_*a*_ = *w*_*b*_ = *w*_*c*_*i*__. This also applies for weights related to constraints *d*_*u*_. However, independent weights can be assigned for the position and orientation components of a constraint.

### 3.6. Initialization of the Kinematic Chains

To accurately estimate the limb posture, it is required to properly couple the human and exoskeleton kinematic models. To do so, we require to correctly position the end-effectors of the human kinematic model with respect to the arm, forearm, and hand coordinate systems. These end-effectors must be positioned such that they are able to move together with the coordinate systems of the fixations of the exoskeleton model (targets). Notice that the position of the end-effectors with respect to the links of the human model changes according to the actual patient and exoskeleton dimensions.

**Figure 6:**
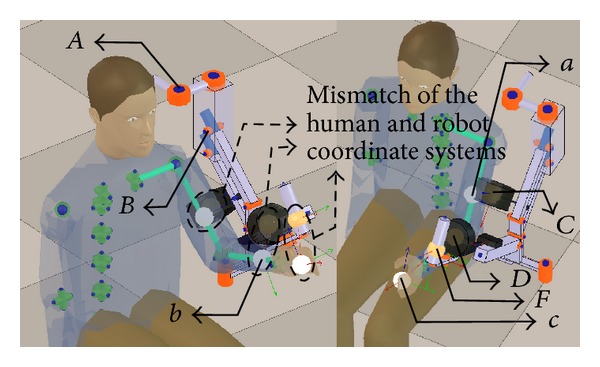
State of the kinematic chains before the initialization process (notation in Table 1).

Figure 6 depicts a state in which the human and exoskeleton models are decoupled. The correct position and orientation of the coordinate systems of the end-effectors of the human model have not been calculated, and, therefore, they do not match the position and orientation of the exoskeleton's fixations coordinate systems.

The initialization of the kinematic chains requires a reference pose of the exoskeleton in which (a) the human joints angles can be determined accurately and (b) the exoskeleton's fixations undergo negligible deformation, reducing the uncertainty about the position of the human model end-effectors.

The pose of the exoskeleton that meets the mentioned requirements is the one in which the flexion/extension of the shoulder and elbow take place in the sagittal plane (Figure 6). In this pose, the position of the human GH joint with respect to the exoskeleton base can be easily determined because the joints of the spine and shoulder complex are in their rest position.

The coupling process involves the following steps.

1. Position the exoskeleton model such that the joint *j*_*R*_0__ lies above the human GH joint. Adjust the height of the exoskeleton model such that *j*_*R*_2__ is at the level of the human GH joint. These instructions are prescribed by the manufacturer of exoskeleton to use it with the actual patient.
2. Compute the arm flexion and abduction angles such that the arm passes through the origin of the arm fixation coordinate system. Adjust the origin of the arm end-effector coordinate system to match the origin of the arm fixation.
3. With the position of the elbow joint defined, compute the elbow flexion and the GH internal rotation angles such that the forearm passes through the origin of the exoskeleton forearm fixation. Adjust the origin of the forearm end-effector coordinate system to match the origin of the forearm fixation.
4. Compute the wrist extension angle such that the human hand is able to grasp the exoskeleton's hand grip. Adjust the hand end-effector to match the position of the Armeo's end-effector at the hand grip.
5. Calculate the forearm pronation/supination angle such that the wrist's extension/flexion axis matches the orientation of the Armeo's hand grip longitudinal axis with respect to the human forearm pronation/supination axis.
6. Adjust the human forearm and hand end-effector coordinate systems to match the orientation of the forearm and Armeo's end-effector coordinate systems, respectively.

**Figure 7:**
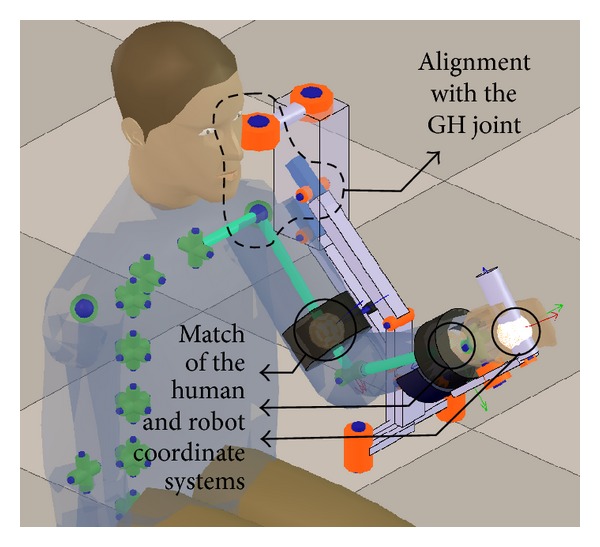
Result of the initialization procedure.

The result of the initialization process is depicted in Figure 7.

## 4. Implementation

To implement the proposed method the virtual robot experimentation platform (V-REP) was used [55], which is an open source robotics simulator. V-REP provides tools to easily and efficiently create kinematic models of rigid multibody systems and to solve IK problems. Using the simulator, a scene was created, which contains both the human upper body and Armeo kinematic models (Figures 2 and 3). The weights of the human kinematic model were adjusted (Section 3.5.2) and the simulator's IK module was configured to include the kinematic constraints (Section 3.4).

The source code of the simulator was compiled, modified, and integrated into our rehabilitation platform. Custom classes and functions were programmed to allow easy data exchange among the Armeo, the rehabilitation game platform, and the IK module of the simulator.

The limb posture estimation process consists of the following steps.

1. Obtain the angles of the Armeo's joints by using hardware and software interfaces provided by HOCOMA AG [47].
2. Use the obtained angles to update the joints angles of the Armeo's kinematic model in the simulator.
3. Retrieve the angles of the joints of the human model computed by the simulator's IK module.

Computing the inverse kinematics of our upper limb kinematic model, once the Armeo model is updated in the simulator with the real joint measurements of the exoskeleton, takes less than 4 ms on a 2.13 Ghz dual-core CPU. Therefore, the implemented method is suitable for real-time posture estimation without using high-performance hardware.

After the joint estimates are produced, we use them to update the patient avatar in VR games. We also store them in a database for a posterior patient assessment.

**Figure 8:**
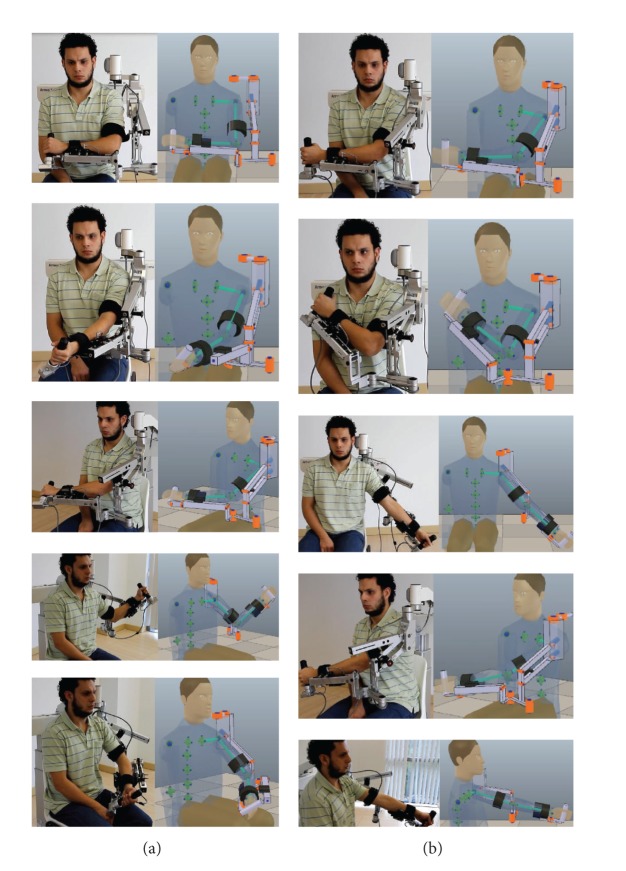
Test subject in parallel with estimations of his posture in the simulator. (a) shows reaching exercises and (b) shows extreme region exercises.

Figure 8 presents a user test of the limb posture estimation algorithm feeding the Armeo kinematic model in the simulator (in real time) with the Armeo Spring joint positions measured by its encoders. This figure presents the posture of the test subject and Armeo Spring in parallel with estimations of the user posture in the simulator. The test subject performed

- reaching exercises, in which the subject recreated the postures of his arm to reach and grab objects that are close to his body (Figure 8(a)). These exercises are frequently practiced during the arm rehabilitation;
- extreme region exercises, in which the subject positioned his hand in the boundaries of his arm workspace (Figure 8(b)). These exercises are challenging for the subject and are less likely to occur during the therapies due to the exercises difficulty.

### 4.1. VR Games

Currently, we have implemented two types of games for the robotic-assisted upper limb rehabilitation therapy. The first type of games focuses on the rehabilitation of reaching movements. The second type of games addresses the rehabilitation of analytic movements of the GH, elbow, and wrist joints.

#### 4.1.1. Reaching Rehabilitation

Reaching rehabilitation is performed by training the movements that are required to reach and grasp objects with the hand. These exercises involve several joints of the upper limb, and, therefore, they are considered complex.

**Figure 9:**
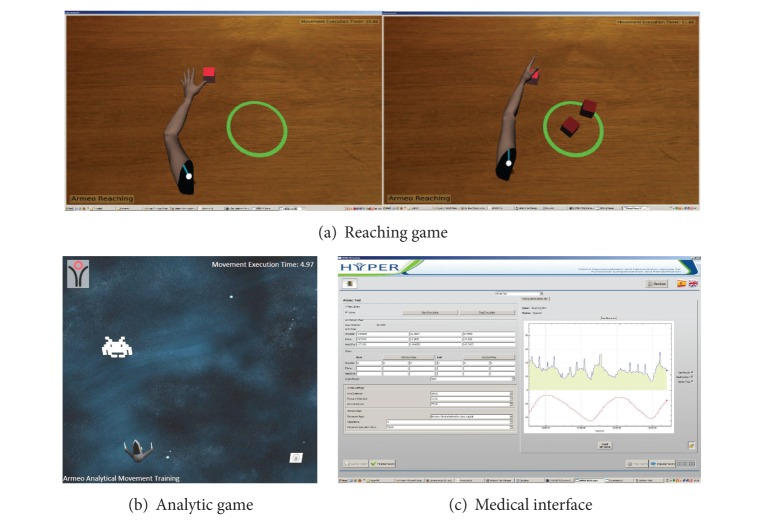
Games and medical interface.

To train these exercises, we have programmed a game in which the patient controls the movement of a virtual human arm by moving his own arm (Figure 9(a)). The target of the patient is to reach a specific object (e.g., cube) in the scene, grab it, and bring it to a releasing area (e.g., green circle).

#### 4.1.2. Analytic Movements Rehabilitation

According to motor learning theories, the training of analytic movements constitutes the first step into learning complex motor tasks. In such a step, simple movements involving few DOFs of the limb are practiced [56–58].

For this scenario, we have programmed a game (Figure 9(b)) in which the patient controls the position of a spaceship, along the horizontal axis of the screen, by performing 1-DOF movements with the wrist, elbow, or GH joint. The target of the game is that the patient positions the spaceship under an alien that moves along a vertical path from the top to the bottom of the screen. When the position of the spaceship is correct, it fires a gun and destroys the alien.

For both games, the limitations of the mobility of the patient are identified in a calibration phase, guarantying that the target of the games is properly located. Other game parameters (number of executions, max execution time per task, target size, etc.) are adjusted through the medical interface (Figure 9(c)). The medical interface allows the physician to select the games for the training, configure its parameters, and review metrics related to the performance of the patient during a game.

The VR games were programed with the OpenSceneGraph API [59], which allows animating deformable virtual objects and creating scenes with dynamic simulations using the Bullet Physics package. The graphic rendering of the VR game runs at 30 frames per second using a ATI Radeon HD 4600 GPU, which is a midrange graphic card.

During the therapy, the patient sees the VR scene. The kinematic models are used for IK computations and they are not displayed.

## 5. Evaluation

In order to determine the accuracy of our developed method, the joint angles of 4 voluntary healthy male test subjects (average age 34 years) were measured by using an optical tracking system and compared with the angles obtained from our posture estimation algorithm during the execution of typical (in this case, analytic movements) robotic-assisted rehabilitation exercises. As discussed in Section 4.1.2, the rehabilitation of analytic movements is a necessary step before addressing the rehabilitation of complex motor tasks.

The specific exercises performed by the test subjects were

1. wrist flexion/extension (WFE),
2. elbow flexion/extension (EFE),
3. forearm pronation/supination (FPS),
4. simultaneous elbow flexion/extension and forearm pronation/supination (SEFEFPS).

The evaluation of our method has been conducted without performing any previous setting or automatic adjustment of the weights or other parameters of the approach in order to reduce the estimation errors. However, algorithm training might be used in the future to improve the method's performance.

### 5.1. Measurement of the Upper Limb Joint Angles

A detailed explanation of the method that was used to measure the human joint angles would merit an additional manuscript. Nevertheless, a basic description of this method is provided next.

In order to measure the limb joint angles of the test subject, we use a Polaris Spectra optical tracking system (OTS) [60]. In order to track the limb movements, it is necessary to install on test subject limb a set of rigid bodies with passive markers. By detecting these passive markers (reflective spheres), the OTS is able to compute the position and orientation of each rigid body.

One rigid body (reference rigid body) is used as the coordinate system of reference for the measurements of the OTS. The position and orientation of the other rigid bodies (mobile rigid bodies) are computed with respect to the reference rigid body.

**Figure 10:**
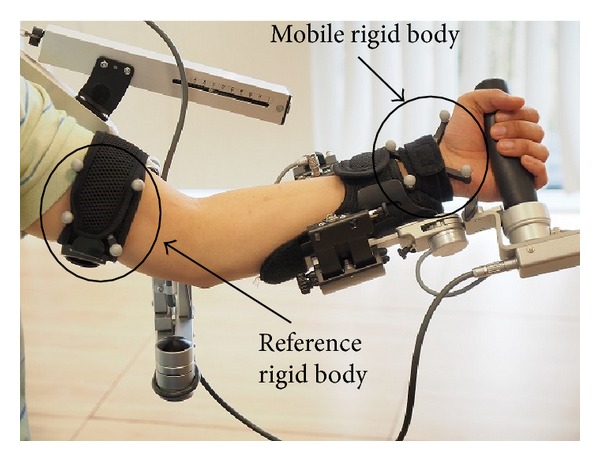
Setup for the quantitative assessment of the estimation errors in elbow flexion/extension exercise.

The reference and mobile rigid bodies are installed on different arm segments (i.e., upper arm, forearm, and hand) according to the joint angle to be measured.  Table 2 shows the installation of the reference and mobile rigid bodies for each of the joint angles that we measured.  Figure 10 shows the configuration of the rigid bodies to measure the elbow flexion/extension angle.

In order to measure the human joint angles, we have adapted the method presented in [61], which is originally proposed to be used with IMMSs, to implement it by using an OTS. In [61] it is proposed to measure the joint angles by following the next steps.

1. Compute a reference coordinate system for the joint of interest. A subset of the axes of the resulting coordinate system match the axes of rotation of the joint. The position and orientation of the joint coordinate system are defined with respect to the reference rigid body.
2. Compute the orientation of the mobile rigid body with respect to the joint coordinate system.
3. Compute the joint angles that result from rotations of the mobile rigid body by using Euler-angles decomposition. The rotations of the mobile rigid body are caused by the exercising of the subject joint.

To build an orthogonal right-handed coordinate system of reference for the joint, we identify each axis of rotation of the joint, as proposed in [61].

To identify each rotation axis of the joint, we use the instant helical axis method described in [62]. A rotational axis of the joint is computed from the kinematic data of the mobile rigid body while the subject performs 1-DOF movements of the joint.

In contrast to the proposal presented in [61] to compute the wrist joint coordinate system, we build this coordinate system by identifying only the flexion/extension axis, given that the ulnar/radial deviation cannot be trained with the Armeo Spring.

*Accuracy of the Limb Joint Angles Measurement Method*. In motor rehabilitation,* goniometry* is the standard method to measure the angle at the patient joints. This is a manual method, and, therefore, its efficacy depends on the examiner experience [63]. One of the limitations of this method is that it provides a resolution (minimal detectable change) in measuring limb joint angles of about 8 degrees [64]. In other words, this method should not be used to measure angles smaller than 8 degrees because in those cases measurements present large uncertainty.

Alternative approaches to measure the patient limb joint angles are IMMS-based methods. One of the methods that provide better accuracy than goniometry is presented in [35]. This method provides a measurement accuracy characterized by a RMSE of less than 3.6 degrees. The authors of the mentioned work conclude that this accuracy is proper for measuring elbow and shoulder angles of clinical relevance in ambulatory settings.

In tests with an artificial 1-DOF joint, the method to measure the limb joint angles that we have adapted from [61] allowed us to estimate the joint angle with a RMSE smaller than 1 degree. According to a comparison with the accuracy provided by the reviewed methods, we conclude that the method proposed by [61] to measure the limb posture is valid to determine the accuracy of our proposed limb posture estimation method.

### 5.2. Protocol

Table 3 summarizes the main features of the evaluation that we have conducted.

For each trial of the evaluation exercises we performed the following steps.

1. Compute the joint coordinate system corresponding to the evaluation exercise (Section 5.1).
2. Instruct the subject to perform the corresponding evaluation exercise until the number of desired joint angle measurements is taken.
3. Compute the RMSE in the estimation of each joint angle by comparing the measured angle with the estimation provided by our algorithm.
4. Compute the ROM of the subject movements from the measured angles.

During the execution of the evaluation exercises the amplitude, speed and the number of cycles of the movements in each trial were left to the discretion of each test subject. In the evaluation, the VR games were not executed, given that they are not necessary to assess the accuracy of the posture estimation algorithm. Furthermore, in this way the influence of the VR games on the subject movement amplitude, speed, and repetitions is avoided, which derives a richer variety of movement features in the evaluation exercises.

However, it is worth mentioning that the joint limits of the exoskeleton, the need to avoid occlusions of the passive markers on the rigid bodies attached to the test subject, and the limited detection volume of the OTS do constrain the subject's movements.

## 6. Results and Discussion

In this section, we present the results of the experiments described in Section 5. Tables 4, 6, 7, and 8 (angles expressed in degrees) present the average RMSE obtained in the estimation of the angle of interest by using our proposed algorithm. Each table presents the average ROM of the movement performed by each test subject. The average RMSE and ROM metrics mentioned previously are obtained from the 4 trials that each subject performed for each exercise. The last row in the tables presents the average values of each of the computed metrics for all subjects.

N.B.: in this section we compare our results against freely moving subject cases reported in the literature. We resort to such free movement cases since we found no reports concerning estimations errors of the wrist or elbow angles in limbs constrained with exoskeletons.

### 6.1. Wrist Flexion/Extension

Table 4 presents angle estimation statistics for wrist flexion and extension. The ROM exercised by the subjects presents small variability and seems not to correlate with RSME. However, we did observe that subjects 1 and 2 performed slow movements while subjects 3 and 4 moved fast. Such a difference reflects on the RMSE values.

**Figure 11:**
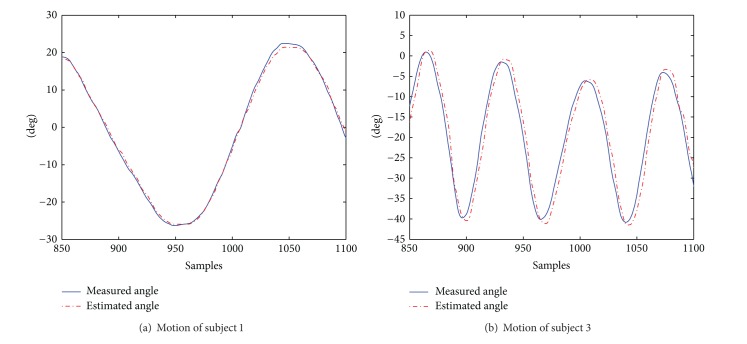
Motion patterns of subjects 1 and 3 during a trial of wrist flexion/extension.

To elaborate this point, we present in Figure 11 the history of the measured versus estimated angle, for subjects 1 and 3. The sampling span is 250 (approx. 3.75 seconds). The motion features of the movements shown in Figure 11 are summarized in Table 5. In such table, the time delay aspect refers to the time delay that the estimations provided by our algorithm present with respect to the measured angles. The time delay is larger when the subject moves fast. This causes the increment in the RMSE estimation values.

These results suggest that the response speed of our algorithm, given a change in the Armeo joint angles caused by the movement of the human subject, allows providing better estimates when the subject moves slowly (as in rehabilitation therapy). In our algorithm, the response speed largely depends on the damping constant used in the DLS method to solve the limb's IK. By using a smaller damping constant in the DLS method, the response speed can be improved, sacrificing some stability.

Nevertheless, the average RMSE obtained for all subjects shows a better performance of our method with respect to [39], an optimization-based approach which presents errors around 3.5 degrees. Compared to [36], which presents a IMMS-based method to estimate the wrist angles with a RMSE of less than 3 degrees, our results are slightly better.

### 6.2. Elbow Flexion/Extension

**Figure 12:**
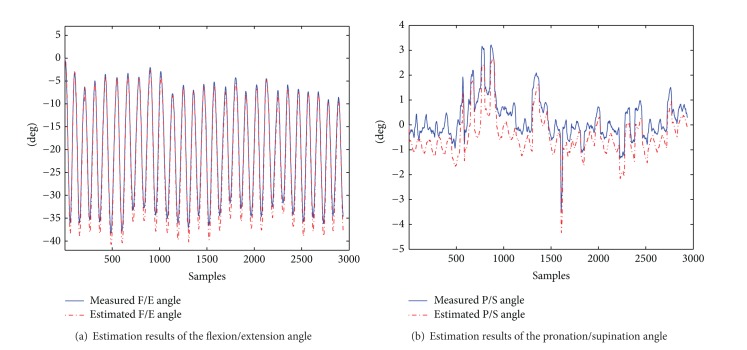
Estimation results of the elbow angles during flexion/extension for trial of subject 2.

In flexion and extension of elbow (Figure 12, Table 6), involuntary movement along the pronation/supination axis is not avoided. Therefore, small excursions in this DOF were observed.

For all subjects, our method overestimates the amplitude of rotational movements about the flexion/extension axis, when compared against the measured values (see Figure 12(a) for subject 2).

Our method performs better than the one in [39], in which the reported mean error in estimating the flexion/extension angle is approximately 14 degrees. Compared to the approach in [35], which uses a IMMS-based method and presents a RMSE of 3.6 degrees in estimating elbow and shoulder angles, our method also presents better performance.

We include in Table 6 the estimation statistics for pronation/supination angle in order to illustrate the performance of our method with small angular displacements.  Figure 12(b) displays the estimation and measurement of pronation/supination angle for a trial of subject 2. In this figure, we observe that there is an underestimation of the angle. However, it must be taken into account that estimation errors for small ROMs are in the same order of the measurement method accuracy (RMSE 1 degree).

### 6.3. Forearm Pronation/Supination

**Figure 13:**
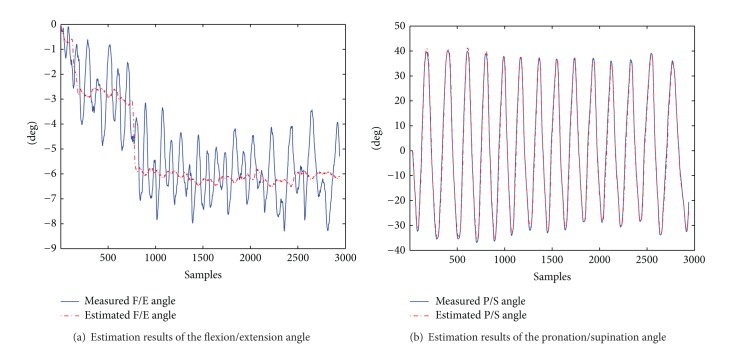
Estimation results of the elbow angles during pronation/supination for a trial of subject 1.

Table 7 and Figure 13 show the statistics of our method for forearm pronation/supination angle estimation. We remark that motion in the elbow flexion/extension axis may occur during the forearm pronation/supination exercise. Therefore, we also report (in Table 7 and Figure 13) the estimation results for the small angular movements around the flexion/extension axis.

The average RMSE in the estimation of the pronation/supination DOF of our method presents an accuracy similar to the one of [35] (RMSE 3.6 degrees).

Figure 13 shows the elbow angles estimation results for a trial of the FPS exercise of subject 1.  Figure 13(a) shows that estimations in the flexion/extension DOF, in which small movements were performed, do not present the oscillations of the measured angle (RMSE 1.175 degrees). On the other hand, Figure 13(b) shows that estimations of the pronation/supination angle are very close to the measured values.

For the pronation/supination angle, the worse estimations were obtained for subject 4, who performed short but very fast movements, affecting the estimation accuracy as described in Section 6.1.

According to results presented here and in Section 6.2, it seems that for small movements the estimation approach is slightly more sensitive to movements in the pronation/supination DOF than on the flexion/extension DOF.

### 6.4. Simultaneous Elbow Flexion/Extension and Forearm Pronation/Supination

The objective of this exercise is to evaluate how simultaneous movements of both DOFs of the elbow affect the angle estimations for this joint. The results are presented in Table 8. In this table, it is shown that, for both elbow DOFs, the average of the RMSE for all the subjects is similar to the one presented in [35] (RMSE 3.6 degrees).

This result also suggests that, during the performance of a functional rehabilitation exercise, such as reaching, in which simultaneous flexion/extension and pronation/supination movement are necessary, the accuracy of the estimations would remain in an adequate range.

**Figure 14:**
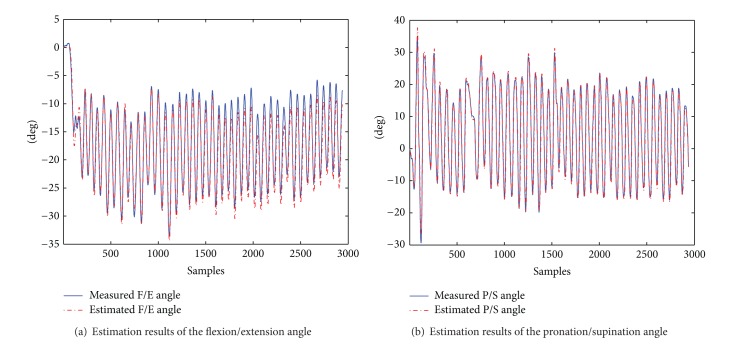
Estimation results of the elbow angles during simultaneous flexion/extension and pronation/supination for a trial of subject 4.

Figure 14 presents the estimation results of a trial of this exercise of subject 4. In this figure, it can be observed that estimations follow closely the measured angles.

## 7. Conclusions and Future Work

This paper presents a method that can be applied to estimate the posture of the human limbs during the interaction with exoskeletons by solving the limb IK problem extended with the kinematic constraints of the exoskeleton fixations on the limb. The few approaches in the literature that deal with limb posture estimation in a robotic-assisted scenario are specifically designed to estimate the arm posture. In contrast, the method that we propose provides a general formulation, which is not specific to any human limb or exoskeleton. Our method is based on inverse kinematics and it can be implemented using standard robotics libraries.

In this paper, we have also shown the implementation of the method to provide upper limb posture estimations, in real time, using the Armeo Spring. We have also presented the use of the resulting limb postures estimations in the animation of avatars in VR rehabilitation games.

We have evaluated the accuracy of the estimations of our method during the performance of analytic rehabilitation exercises of the wrist and elbow. The obtained results show that our approach presents an accuracy that is better than the one provided by goniometry, which is the traditional method to measure the patient angles in motor rehabilitation. Compared to the accuracy provided by IMMSs-based methods, which are considered enough accurate to measure clinical relevant limb joint angles in nonrobotic-assisted scenarios, we have obtained very similar results.

Based on the mentioned results, we conclude that our approach can be used to (a) provide an estimation of the pose of the human upper limb with enough accuracy to be used for avatar animation in VR games and (b) obtain the kinematic data for the patient assessment during analytic training of the elbow and wrist.

Future work includes (a) the exploration of other approaches to model the flexible fixations of the exoskeleton, (b) the definition of a set of weights for the human model joints that represent the movement features of a set of human subjects, and (c) a quantitative assessment of the performance of our method in a functional rehabilitation scenario.

## Figures and Tables

**Table 1:** Glossary related to the Figure 6.

Symbol	Description
*A*	*j*_*R*_0__
*B*	*j*_*R*_2__
*C*	Arm fixation coordinate system
*D*	Forearm fixation coordinate system
*F*	Armeo hand grip coordinate system
*a*	Human arm end-effector coordinate system
*b*	Human forearm end-effector coordinate system
*c*	Human hand end-effector coordinate system

**Table 2:** Installation of the reference and mobile rigid bodies in the evaluation.

Angle to measure	Reference rigid body installed on	Mobile rigid body installed on
WFE	Forearm	Hand
EFE	Upper arm	Forearm
FPS	Upper arm	Forearm

**Table 3:** Summary of main features of the evaluation tests.

Number of test subjects	Number of exercises performed by each test subject	Number of trials per exercise	Joint angles measurements per trial
4	4 (WFE, EFE, FPS, and SEFEFPS)	4	2960 at 66.6 Hz

**Table 4:** Estimation errors in wrist flexion/extension exercise (units in degrees).

Subject	Average WFE RMSE	Average WFE ROM
1	1.137	53.389
2	1.432	54.824
3	3.282	63.869
4	3.555	53.977

Average	2.351	56.265

**Table 5:** Motion features for subjects 1 and 3 in WFE exercise.

Aspect	Subject 1	Subject 3
Average angular speed (deg/s)	26	82
Time delay (ms)	15	60

**Table 6:** Estimation errors in elbow flexion/extension exercise (units in degrees).

Subject	Av. EFE RMSE	Av. EFE ROM	Av. FPS RMSE	Av. FPS ROM
1	1.636	36.948	0.980	4.148
2	1.553	33.897	1.408	4.921
3	2.815	49.333	2.187	5.216
4	4.381	36.442	1.128	7.160

Average	2.596	39.150	1.426	5.361

**Table 7:** Estimation errors in forearm pronation/supination exercise (units in degrees).

Subject	Av. EFE RMSE	Av. EFE ROM	Av. FPS RMSE	Av. FPS ROM
1	1.221	5.799	1.965	70.453
2	1.799	7.395	2.639	48.500
3	1.627	9.691	4.147	90.527
4	1.132	2.459	4.568	37.717

Average	1.445	6.336	3.330	61.799

**Table 8:** Estimation errors in simultaneous elbow flexion/extension and forearm pronation/supination exercise (units in degrees).

Subject	Av. EFE RMSE	Av. EFE ROM	Av. FPS RMSE	Av. FPS ROM
1	2.224	35.762	2.707	59.878
2	2.773	40.837	3.037	58.441
3	5.212	47.850	4.429	55.228
4	2.679	36.654	2.158	59.673

Average	3.222	40.276	3.083	58.305

## Nomenclature

Clavicle: One of the bones of the shoulder girdle. It is located at the root of the neck
DLS: Damped least squares
DOF: Degree of freedom
EFE: Elbow flexion/extension
FPS: Forearm pronation/supination
GH: Glenohumeral
Humerus: Upper arm bone
IK: Inverse kinematics
IMMSs: Inertial and magnetic measurement systems
OTS: Optical tracking system
RMSE: Root mean square error
ROM: Range of motion
Scapula: One of the bones of the shoulder girdle. It connects the humerus with the clavicle
SEFEFPS: Simultaneous EFE and FPS
VR: Virtual reality
V-REP: Virtual robot experimentation platform
WFE: Wrist flexion/extension
*v*: Total number of constraints of the IK problem (*v* ∈ *N*)
*e*: IK error vector (*e* ∈ *R* ^*v*^)
*k*: Total DOFs of the human kinematic model (*k* ∈ *N*)
*n*: Total DOFs of the exoskeleton kinematic model (*n* ∈ *N*)
*Z*: Jacobian matrix of the IK problem (*Z* ∈ *R* ^*v*×*k*^)
*I*: *v* × *v* identity matrix
*W*_*q*_: Diagonal matrix of joints weights (*W*_*q*_ ∈ *R* ^+^^*k*×*k*^)
*W*_*e*_: Diagonal matrix of constraints weights (*W*_*e*_ ∈ *R* ^+^^*v*×*v*^)
*q*_*H*_*t*__: Vector of joint angles of the human kinematic model in instant *t* (*q*_*H*_*t*__ ∈ *R* ^*k*^)
*q*_*R*_*t*__: Vector of joint angles of the exoskeleton kinematic model in instant *t* (*q*_*R*_*t*__ ∈ *R* ^*n*^)
*α*: Damping factor of DLS method (*α* ∈ *R* ^+^).

## References

[1] Krakauer JW (2006). Motor learning: its relevance to stroke recovery and neurorehabilitation. *Current Opinion in Neurology*.

[2] Adamevich SV, Merians AS, Boian R (2005). A virtual reality-based exercise system for hand rehabilitation post-stroke. *Presence: Teleoperators and Virtual Environments*.

[3] Riener R, Nef T, Colombo G (2005). Robot-aided neurorehabilitation of the upper extremities. *Medical & Biological Engineering & Computing*.

[4] De Mauro A, Carrasco E, Oyarzun D, Gulrez T, Hassanien A (2012). Advanced hybrid technology for neurorehabilitation: the HYPER project. *Advances in Robotics and Virtual Reality*.

[5] Jarrassé N, Morel G (2010). On the kinematic design of exoskeletons and their fixations with a human member. *Robotics: Science and Systems*.

[6] Kim H, Miller LM, Li Z, Roldan JR, Rosen J Admittance control of an upper limb exoskeleton—reduction of energy exchange.

[7] Guidali M, Duschau-Wicke A, Broggi S, Klamroth-Marganska V, Nef T, Riener R (2011). A robotic system to train activities of daily living in a virtual environment.. *Medical & biological engineering & computing*.

[8] Frisoli A, Procopio C, Chisari C (2012). Positive effects of robotic exoskeleton training of upper limb reaching movements after stroke. *Journal of NeuroEngineering and Rehabilitation*.

[9] Gilliaux M, Lejeune T, Detrembleur C, Sapin J, Dehez B, Stoquart G (2012). A robotic device as a sensitive quantitative tool to assess upper limb impairments in stroke patients: a preliminary prospective cohort study. *Journal of Rehabilitation Medicine*.

[10] Fasoli SE, Krebs HI, Stein J, Frontera WR, Hogan N (2003). Effects of robotic therapy on motor impairment and recovery in chronic stroke. *Archives of Physical Medicine and Rehabilitation*.

[11] Brewer BR, McDowell SK, Worthen-Chaudhari LC (2007). Poststroke upper extremity rehabilitation: a review of robotic systems and clinical results. *Topics in Stroke Rehabilitation*.

[12] Teasell RW, Kalra L (2004). Whats new in stroke rehabilitation. *Stroke*.

[13] Epelde G, Valencia X, Ardanza A Virtual arm representation an d multimodal monitoring for the upper limb robot assisted teletherapy.

[14] Lo HS, Xie SQ (2012). Exoskeleton robots for upper-limb rehabilitation: state of the art and future prospects. *Medical Engineering and Physics*.

[15] Shumway-Cook A, Woollacott MH (2007). *Motor Control: Translating Research into Clinical Practice*.

[16] Schmidt RA, Lee T Motor Control and Learning, 5E.

[17] Broeren J, Sunnerhagen KS, Rydmark M (2007). A kinematic analysis of a haptic handheld stylus in a virtual environment: a study in healthy subjects. *Journal of NeuroEngineering and Rehabilitation*.

[18] Chaffin DB (2007). Human motion simulation for vehicle and workplace design. *Human Factors and Ergonomics in Manufacturing & Service Industries*.

[19] Faraway J, Reed MP (2007). Statistics for digital human motion modeling in ergonomics. *Technometrics A: Journal of Statistics for the Physical, Chemical and Engineering Sciences*.

[20] Baerlocher P, Boulic R (2004). An inverse kinematics architecture enforcing an arbitrary number of strict priority levels. *Visual Computer*.

[21] Kim H, Miller LM, Byl N, Abrams GM, Rosen J (2012). Redundancy resolution of the human arm and an upper limb exoskeleton. *IEEE Transactions on Biomedical Engineering*.

[22] Jung ES, Shin Y (2010). Two-handed human reach prediction models for ergonomic evaluation. *Human Factors and Ergonomics in Manufacturing & Service Industries*.

[23] Xiang Y, Arora JS, Abdel-Malek K (2012). 3D human lifting motion prediction with different performance measures. *International Journal of Humanoid Robotics*.

[24] Ma L, Zhang W, Chablat D, Bennis F, Guillaume F (2009). Multi-objective optimisation method for posture prediction and analysis with consideration of fatigue effect and its application case. *Computers & Industrial Engineering*.

[25] Mi Z, Yang J, Abdel-Malek K (2009). Optimization-based posture prediction for human upper body. *Robotica*.

[26] Li Z, Kim H, Milutinović D, Rosen J (2013). Synthesizing redundancy resolution criteria of the human arm posture in reaching movements. *Redundancy in Robot Manipulators and Multi-Robot Systems*.

[27] Yang J, Marler RT, Beck S, Abdel-Malek K, Kim J (2006). Real-time optimal reach-posture prediction in a new interactive virtual environment. *Journal of Computer Science and Technology*.

[28] Gragg J, Yang J Posture reconstruction-some insights.

[29] Yang J, Rahmatalla S, Marler T, Abdel-Malek K, Harrison C Validation of predicted posture for the virtual human santos.

[30] Yang J, Marler RT, Kim H, Arora JS, Abdel-Malek K Multi-objective optimization for upper body posture prediction.

[31] Pasciuto I, Ausejo S, Celigüeta JT, Suescun Á, Cazón A (2014). A comparison between optimization-based human motion prediction methods: data-based, knowledge-based and hybrid approaches. *Structural and Multidisciplinary Optimization*.

[32] Xiang Y, Arora JS, Abdel-Malek K (2012). Hybrid predictive dynamics: a new approach to simulate human motion. *Multibody System Dynamics*.

[33] Jarrasse N, Crocher V, Guillaume M A method for measuring the upper limb motion and com puting a compatible exoskeleton trajectory.

[34] Perry JC, Rosen J, Burns S (2007). Upper-limb powered exoskeleton design. *IEEE/ASME Transactions on Mechatronics*.

[35] Cutti AG, Giovanardi A, Rocchi L, Davalli A, Sacchetti R (2008). Ambulatory measurement of shoulder and elbow kinematics through inertial and magnetic sensors. *Medical and Biological Engineering and Computing*.

[36] Zhou H, Hu H (2010). Reducing drifts in the inertial measurements of wrist and elbow positions. *IEEE Transactions on Instrumentation and Measurement*.

[37] Yang J, Abdel-Malek K, Nebel K (2005). Reach envelope of a 9-degree-of-freedom model of the upper extremity. *International Journal of Robotics and Automation*.

[38] Zou Q, Zhang Q, Yang J Determining weights of joint displacement objective function for standing reach tasks.

[39] Zou Q, Zhang Q, Yang J An alternative formulation for determining weights of joint displacement objective function in seated posture prediction.

[40] Gragg J, Yang J, Howard B (2012). Hybrid method for driver accommodation using optimization-based digital human models. *Computer Aided Design*.

[41] Hill AM, Bull AMJ, Wallace AL, Johnson GR (2008). Qualitative and quantitative descriptions of glenohumeral motion. *Gait & Posture*.

[42] Maurel W, Thalmann D (2000). Human shoulder modeling including scapulo-thoracic constraint and joint sinus cones. *Computers & Graphics*.

[43] Holzbaur KRS, Murray WM, Delp SL (2005). A model of the upper extremity for simulating musculoskeletal surgery and analyzing neuromuscular control. *Annals of Biomedical Engineering*.

[44] Ambrósioa J, Quental C, Pilarczyk B, Folgado J, Monteiro J Multibody biomechanical models of the upper limb.

[45] Kim S, Kim D, Chae S (2009). Musculoskeletal upper limb modeling with muscle activation for flexible body simulation. *International Journal of Precision Engineering and Manufacturing*.

[46] Denavit J, Hartenberg RS (1955). A kinematic notation for lower-pair mechanisms based on matrices. *Transactions of the ASME, Journal of Applied Mechanics*.

[47] HOCOMA AG (2013). *Armeo Spring—Functional Arm and Hand Therapy for Children*.

[48] Cloutier A, Boothby R, Yang J Motion capture experiments for validating optimization-based human models.

[49] Grassia FS (1998). Practical parameterization of rotations using the exponential map. *Journal of Graphics Tools*.

[50] Rosen J, Perry JC, Manning N, Burns S, Hannaford B The human arm kinematics and dynamics during daily activities-toward a 7 DOF upper limb powered exoskeleton.

[51] Chang PH (1987). Closed-form solution for inverse kinematics of robot manipulators
with redundancy. *IEEE Journal of Robotics and Automation*.

[52] Buss SR http://math.ucsd.edu/~sbuss/ResearchWeb/ikmethods/iksurvey.pdf.

[53] Buss SR, Kim J-S (2005). Selectively damped least squares for inverse kinematics. *Journal of Graphics, GPU, and Game Tools*.

[54] Schinstock DE, Faddis TN, Greenway RB Robust inverse kinematics using damped least squares with dynamic weighting.

[55] http://www.coppeliarobotics.com/.

[56] Shumway-Cook A, Woollacott MH (2012). *Motor Control: Translating Research into Clinical Practice*.

[57] Vereijken B, Richard van Emmerik EA, Whiting HTA, Karl Newell M (1992). Freezing degrees of freedom in skill acquisition. *Journal of Motor Behavior*.

[58] Cano-de-la Cuerda R, Molero-Sanchez A, Carratala-Tejada M (2012). *Teoriasy Modelos de Control y Aprendizaje Motor. Aplicaciones Clinicas en Neurorrehabilitaci on Neurologia*.

[59] OpenSceneGraph Openscenegraph. http://trac.openscenegraph.org/projects/osg/.

[60] NDI (January 2014). *Polaris Family of Optical Tracking Systems*.

[61] de Vries WHK, Veeger HEJ, Cutti AG, Baten C, van der Helm FCT (2010). Functionally interpretable local coordinate systems for the upper extremity using inertial & magnetic measurement systems. *Journal of Biomechanics*.

[62] Veeger HEJ, Yu B Orientation of axes in the elbow and forearm for biomechanical modelling.

[63] de Carvalho RMF, Mazzer N, Barbieri CH (2012). Analysis of the reliability and reproducibility of goniometry compared to hand photogrammetry. *Acta Ortopedica Brasileira*.

[64] Kolber MJ, Fuller C, Marshall J, Wright A, Hanney WJ (2012). The reliability and concurrent validity of scapular plane shoulder elevation measurements using a digital inclinometer and goniometer. *Physiotherapy Theory and Practice*.

